# Advice to the Public Concerning the Manner of Using an Ointment of Corrosive Sublimate in the Cure of the Venereal Disease, and of Other Disorders Which Often Resist the Best Remedies

**Published:** 1783

**Authors:** 


VII. *Avvifo al Pubblico*, &c. i. e. *Advice to the Public concerning the manner of ufing an ointment of corrofive fublimate in the cure of the Venereal difeafe, and of other diforders which often refift the beft remedies. By* Dominic Cirillo, M. D. *Profeffor of the Practice of Phyfic in the Univerfity of Naples*, &c. 8vo. Naples.

THE author of this tract having in the courfe of a long practice made frequent

 ufe ufe of fublimate, both in fubftance and diffolved in fpirit of wine, and having often feen it produce dangerous and even fatal fymptoms, as well as fail in the removal of venereal complaints, was induced to try its effects when applied externally in the following form;

℞ Mercurii fublimati corrofivi in pulv. fubtiliffim. redact. drachmam unam.
Axungiæ porcinæ, unciam unam.
Tritur. fimul diligenter per horas xij ut fiat unguentum.

Of this ointment he directs from half a drachm to two drachms to be rubbed every night on the foles of the feet. He particularly mentions this part, becaufe, if rubbed on the legs, or where the cuticle is thinner, it is liable to produce ulceration. The fame precautions and regimen are to be adopted as in the cure by frictions with the common mercurial ointment.

With this remedy, he tells us, he has fucceeded in a great number of cafes where the ordinary modes of treatment have failed, both in the hofpital for incurables, to which he is phyfician, and in private practice. He has feen it, for inftance, remove different venereal ophthalmia's, pains of the limbs accompanied with fpurious exoftofes, and even venereal fwellings of the knee joint, a comcomplaint which usually bids defiance to every species of mercurial preparation, and which terminates sometimes in a stiff joint, and at others in a dropsy of the joint, or in phthisis in consequence of suppuration. He has likewise found this application singularly efficacious in obstinate sciatica. In cases of this sort he confines the friction to the sole of the foot corresponding with the diseased part. In scrophulous cases, and in enlargement of the epidydimis, he has employed this remedy with equal success.

In every case he recommends a cautious use of it, advising us to begin with small doses, which are to be gradually increased according to the symptoms, age, or constitution of the patient, and other circumstances.

